# Supplementary material for: Machine learning prediction of incidence of Alzheimer’s disease using large-scale administrative health data
Source: NPJ Digit Med. 2020 Mar 26;3:46. doi: 10.1038/s41746-020-0256-0 (PMC7099065; doi:10.1038/s41746-020-0256-0)
Supplement: Supplementary file 1 — Supplementary Information [file 41746_2020_256_MOESM1_ESM.pdf]

Machine Learning Prediction of Incidence of Alzheimer's Disease Using Large-Scale Administrative Health Data

Ji Hwan Park, Han Eol Cho, Jong Hun Kim, Melanie Wall, Yaakov Stern, Hyunsun Lim, Shinjae Yoo, Hyoung Seop Kim, Jiook Cha

Supplementary Materials:

Supplementary Figure 1

Supplementary Table 1-4

Supplementary Figure 1. Medical insurance system dementia medication in Korea.

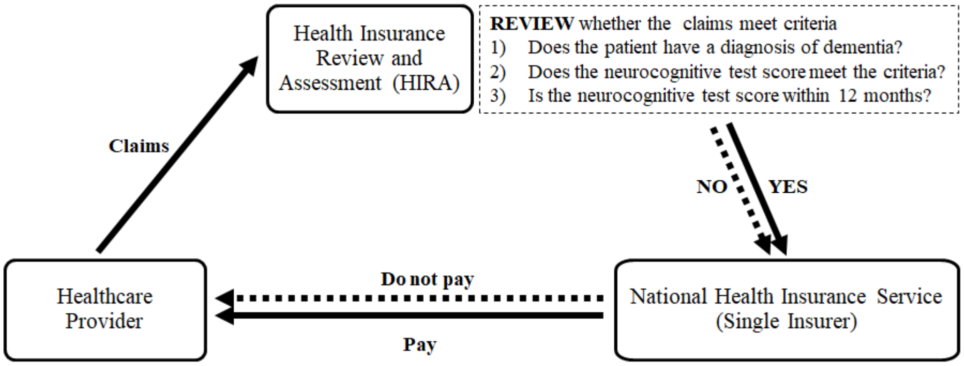

Supplementary table 1. Performance of predictive models trained on EHR by using unbalanced data

	Definite AD (AD codes and dementia prescription)					
	Classifier*	AD/non-AD	AUC	Accuracy	Sensitivity when 90% specificity	Specificity when 90% Sensitivity
0 yr	RF	614/38,710	0.887	0.984	0.687	0.737
1 yr	SVM	672/38,967	0.781	0.980	0.380	0.475
2 yr	SVM	640/38,605	0.739	0.981	0.281	0.400
3 yr	SVM	605/29,983	0.686	0.974	0.227	0.291
4 yr	RF	491/14,196	0.662	0.966	0.000	0.151
	**Probable AD (AD codes)**					
	Classifier*	AD/non-AD	AUC	Accuracy	Sensitivity when 90% specificity	Specificity when 90% Sensitivity
0 yr	RF	2,026/38,710	0.805	0.951	0.240	0.456
1 yr	RF	2,049/38,967	0.730	0.957	0.170	0.338
2 yr	LR	1,892/38,605	0.645	0.931	0.136	0.301
3 yr	LR	1,697/29,983	0.575	0.916	0.085	0.253
4 yr	RF	1,412/14,196	0.602	0.880	0.020	0.018

*best classifiers based on AUC. AUC, area under the curve (based on receiver-operator characteristics LR, logistic regression; RF, random forest; SVM, support vector machine)

Supplementary Table 2. Lengths of EHR (look-back periods) and number of features

	Number of features	Definite AD		Probable AD		Non-AD	
		Average EHR length per subject in days	Average number of non-zero features per subject	Average EHR length per subject in days	Average number of non-zero features per subject	Average EHR length per subject in days	Average number of non-zero features per subject
0 yr	4,894	1936 (1906-1967)	162 (156-167)	2239 (2205-2273)	185 (179-192)	3033 (3028-3038)	176 (174-177)
1 yr	4,722	1851 (1800-1902)	172 (161-182)	1936 (1906-1967)	162 (156-167)	2694 (2690-2698)	164 (163-165)
2 yr	4,622	1571 (1524-1619)	141 (133-149)	1656 (1627-1684)	139 (134-144)	2381 (2378-2384)	151 (150-152)
3 yr	4,494	1666 (1622-1710)	146 (138-154)	1736 (1709-1763)	144 (139-150)	2045 (2042-2047)	135 (134-136)
4 yr	4,353	1736 (1691-1781)	158 (147-169)	1822 (1796-1848)	152 (146-158)	1711 (1708-1714)	116 (114-117)

*The range indicates minimum and maximum

Supplementary table 3. Performance of predictive models using top 20 features selected in logistic regression

Sample	subsequent years of incidence predicted	Classifier	Accuracy	AUC	Sensitivity	Specificity
Definite AD (AD/ non-AD 614/614)	0 yr	LR	0.761	0.805*	0.751	0.772
		RF	0.759	0.805	0.726	0.791
	1 yr	LR	0.722	0.758	0.699	0.746
		RF	0.724	0.772*	0.707	0.741
	2 yr	LR	0.716	0.757	0.664	0.769
		RF	0.720	0.775*	0.666	0.773
	3 yr	LR	0.679	0.706	0.712	0.646
		RF	0.674	0.722*	0.668	0.679
	4 yr	LR	0.720	0.728	0.727	0.713
		RF	0.719	0.739*	0.719	0.719
Probable AD (AD/ non-AD 2026/2026)	0 yr	LR	0.690	0.732*	0.663	0.716
		RF	0.663	0.715	0.605	0.722
	1 yr	LR	0.680	0.720	0.661	0.699
		RF	0.675	0.723*	0.663	0.688
	2 yr	LR	0.680	0.736	0.683	0.677
		RF	0.680	0.737*	0.647	0.713
	3 yr	LR	0.652	0.706*	0.554	0.749
		RF	0.643	0.699	0.692	0.593
	4 yr	LR	0.656	0.708	0.579	0.733
		RF	0.671	0.714*	0.654	0.688

* best performing models based on AUC.
AD, Alzheimer's disease; LR, logistic regression; RF, random forest

Supplementary Table 4. Sociodemographic and Health Profile Variables Use in The Model.

Variables	Type of variable	Explanation
Age	continuous	In years
Sex	binary	0: Female; 1 : Male
Body mass index	continuous	Weight(kg) / (Height*Height)(m2)
Systolic blood pressure	continuous	mmHg
Diastolic blood pressure	continuous	mmHg
Fasting glucose	continuous	mg/dL
Hemoglobin	continuous	Measured from 2009<br>g/dL
Urine protein	ordinal	Measured from 2009<br>1 : negative (-)<br>2 : weak positive (±)<br>3 : positive (1+)<br>4 : positive (2+)<br>5 : positive (3+)<br>6 : positive (4+)
Serum creatinine	continuous	mg/dL
Serum AST	continuous	U/L
Serum ALT	continuous	U/L
r-GTP	continuous	U/L
Family history of liver disease	binary	1 : no<br>2 : yes
Family history of hypertension	binary	
Family history of stroke	binary	
Family history of cardiac disease	binary	
Family history of diabetes mellitus	binary	
Family history of cancer	binary	
Smoking status	continuous	1 : Never smoked<br>2 : Not current smoker but smoked in the past<br>3 : Current smoker
Total smoking period	ordinal	1 : below 5 years<br>2 : 5-9 years<br>3 : 10-19 years<br>4 : 20-29 years<br>5 : over 30 years
Current daily amount of smoking	ordinal	1 : 1~ 12 cigarettes<br>2: 13-24 cigarettes<br>3 : 25~48 cigarettes<br>4 : over 49 cigarettes
Frequency of drinking alcohol	ordinal	1 : almost none<br>2 : 2~3 per month<br>3: 1~2 per week<br>4 : 3~4 per week<br>5 : almost everyday
Amount of alcohol intake in one day	ordinal	1 : below 30g of alcohol<br>2 : below 60g of alcohol<br>3 : below 90g of alcohol<br>4 : over 120g of alcohol
